# Procedural skills and neurobehavioral freedom

**DOI:** 10.3389/fnhum.2014.00449

**Published:** 2014-06-20

**Authors:** Nerea Crespo-Eguílaz, Sara Magallón, Juan Narbona

**Affiliations:** Pediatric Neurology Unit and Department of Education, University of NavarraPamplona, Spain

**Keywords:** attention, central coherence, developmental coordination disorder, implicit memory, operational habit, pragmatics, procedural learning, social communication disorder

## Introduction

Procedural learning (PL) is a part of implicit memory (Shiffrin and Schneider, 1977) and is based on brain subsystems of associative cortex and its connections with basal ganglia and cerebellum (Squire, 1992). PL gives the human individual a gain in freedom: automatic healthy cognitive and motor skills help to save an important amount of conscious work in daily routines and in effortful cognitive and/or motor action (Treisman and Gelade, 1980; Kahneman et al., 1983). In this way, attention can be focused on quick understanding, central coherence awareness, problem-solving processes and social accuracy. Human procedural skills and executively-controlled aspects of action intersect and cooperate with each other (Leisman et al., 2014). Useful procedural automatisms are basically acquired during childhood and youth, but also over the whole course of life, by means of incidental experience and by formal education. PL enhances the natural potentialities (i.e., predispositions) of the agent for a suitable unfolding of his or her operations. From this point of view, acquired automatisms could be included among operational habits in the interface between perceptual-motor and cognitive-volitional human activities.

## Developmental coordination disorder: an expanded view

There is a child population for which operational habit learning is particularly difficult. Clumsiness, disproportionate to general development, is the most evident characteristic of individuals with this developmental condition, which has been labeled *developmental dyspraxia* and, more recently, *developmental coordination disorder* (DCD). At present, the most widely accepted definition of DCD in childhood comes from the Diagnostic and Statistical Manual of Mental Disorders, IVth and 5th editions (APA-American Psychiatric Association, 2000, 2013) and from the International Classification of Diseases-11th edition draft (World Health Organisation, 2013). DCD is essentially regarded as a disturbance of motor coordination, which consequently is substantially below that expected given the child's age and intelligence, but this is not due to a general medical condition (e.g., cerebral palsy) and does not meet the criteria for a pervasive developmental disorder; if mental retardation is present, motor difficulties exceed those expected for the level of mental development. DCD causes disruption of daily living activities and academic achievement. DCD is estimated to affect to 2–8% of schoolchildren (Kadesjö and Gillberg, 1998; Crespo-Eguílaz and Narbona, 2009; Lingam et al., 2009; Missiuna et al., 2011).

Young people with DCD have a characteristic slowness in daily routines. They are disproportionately unskilled not only for motor actions, as can be measured using *ad-hoc* scales and batteries (Bruininks and Bruininks, 2005; Henderson and Sugden, 2007; Wilson et al., 2009) but also for quick perceptual management of complex visuospatial information and motor imagery (Noten et al., 2014). Moreover DCD has a high comorbidity with attention deficit / hyperactivity disorder (ADHD), and with social communication (language pragmatic) disorder (Gillberg, 2003; Crespo-Eguílaz and Narbona, 2009; Crespo-Eguílaz et al., 2012; American Psychiatric Association, DSM-5, 2013; World Health Organisation, IDC-11 draft, 2013). As a consequence, affected children and adolescents typically behave in a naïve manner, and their social use of language is frequently inaccurate (Volden, 2004; Crespo-Eguílaz and Narbona, 2009; Brossard-Racine et al., 2011; Westendorp et al., 2011; Blank et al., 2012). All these impairments have a significant negative impact on activities of daily living, such as, dressing, handwriting, sports, and social exchanges (Blank et al., 2012). Depression, anxiety, and risk of bullying by peers are significantly more frequent in children with DCD and those with comorbid DCD and ADHD vs. typical controls (Zwicker et al., 2012; Missiuna et al., 2014).

## Procedural learning in children with developmental coordination disorder: some experimental evidence

A variety of dysfunctions of neural loops relating prefrontal, secondary premotor and parietal cortices, with basal ganglia and cerebellum, have been proposed (Bo and Lee, 2013; Leisman et al., 2014) to explain the physiopathology of DCD.

In a continuous task with implicit visual sequences, schoolchildren with DCD learn poorly relative to typically developing children. Children with DCD demonstrated a general learning of visuo-perceptive task demands that was comparable to that of controls, but they failed to learn anticipation of implicit visuo-motor sequences. Interestingly, a sequence recall test, administered after the whole task, indicated some awareness of the repeating sequence pattern (Gheysen et al., 2011). By contrast, using the same paradigm, Lejeune et al. (2013) found no evidence of a difference in performance between children with DCD and typically developing children.

In order to assess PL in children with ADHD, DCD and reading learning disorder (RD), Magallón et al. (submitted) tested the children with two implicit / procedural learning tasks using the Purdue pegboard (Gardner and Broman, 1979) and an adaptation of the mirror drawing task (Milner et al., 1968). Participants aged 6–12 years old were classified into four groups matched for gender, age and severity of ADHD symptoms: 19 children with ADHD only, 30 children with DCD+ADHD, 48 children with RD+ADHD, and 90 typically developing children. All participants accomplished three consecutive trials of each of the two tasks and a delayed fourth trial following a verbal interference task. Typical-for-age scoring measures of performance were compared (Student's t) within trials and between groups. The baseline results of the DCD+ADHD group were significantly lower than those of the other groups. Nevertheless, after three repetitions of the two tasks, DCD+ADHD children improved their efficiency and reached that of the baseline of both the non-DCD clinical groups. This learned performance was retained at the delayed fourth trial. However, the percentage improvement obtained by DCD children was lower than that of the other two clinical groups and controls in all the trials.

Another study (Crespo-Eguílaz et al., 2012) addressed the ability of schoolchildren to quickly grasp and verbally explain “nonsense” in complex figurative pictures: a chimeric figure and an absurd scene. Only 11.3% of schoolchildren with DCD and with DCD+ADHD resolved the tasks accurately, whereas 87.5% of controls and ADHD-alone children did these two central coherence function tasks successfully (chi-square test: *p* < 0.01).

As mentioned above, children with DCD+ADHD also usually have difficulties integrating inputs of complex visual or verbal information. As a consequence, they struggle to get the whole picture, miss relevant clues in social contexts, have problems dealing with inference, and fail to make sense of figurative language, jokes, narratives and adapted conversation. These psycholinguistic difficulties are reminiscent of the characteristics of Social (pragmatic) Communication Disorder (SCD) as defined in DSM-5 and in ICD-11 draft. So, we might ask whether DCD is typically comorbid with SCD. An alternative explanation would be that pragmatic difficulties are a part of DCD. To investigate this question, a Spanish translation of the Children's Communication Checklist-CCC (Bishop, 1998) was given to the parents of children aged 6–12 years who were divided into five groups: those with DCD+ADHD, those with ADHD only, those with SCD, those with high functioning autism spectrum disorder (HFASD), and those with typical development (Narbona et al., in press). The five groups were matched for mental age and gender. The results suggest that communication difficulties in children with DCD+ADHD are qualitatively different, more severe and have a larger impact on social relationships than those shown by children with ADHD only. On the other hand, the pragmatic difficulties in children with DCD+ADHD are milder than those defining SCD and HFASD. Moreover the HFASD group showed unusual, restricted and stereotyped interests. In contrast, DCD+ADHD and SCD groups do not have a characteristic restriction of interests, and their basic social motivation and abilities are preserved, apart from the linguistic difficulties. These results are in accordance with recent research reviews (Gibson et al., 2013; Norbury, 2014).

Pragmatic difficulties may be present in children with ADHD, developmental coordination disorder, autism spectrum disorders, Williams syndrome, spina bifida with hydrocephalus, cerebral palsy, etc. (Holck et al., 2009); thus pragmatic difficulties can be either a component of several large behavioral phenotypes or an isolated communication disorder (i.e., SCD, as it has been recently proposed in DSM-5 and in ICD-11 draft). Given that children with DCD most frequently have pragmatic difficulties, it would seem that these are not comorbid but constitute a component of DCD related to the failure to grasp visuospatial clues useful in evaluating social appropriateness. In contrast, pragmatic communication difficulties of autistic persons are included in their social/intersubjective pervasively disordered abilities (Norbury, 2014).

## Discussion and concluding remarks

We propose that the core dysfunction in DCD affects procedural learning. PL deals not only with motor skills but also with fast perceptive integration, cognitive routines and socially accurate habits. As a consequence, children with DCD are characterized by slowness not only for motor tasks but also for awareness of relevant cognitive and social clues, which causes difficulties in contextualizing information and in social relationships with peers. Children with DCD do have normal intersubjective skills and a normal desire to communicate with other people, in contrast to children with autistic spectrum disorders (Norbury, 2014). The above-mentioned experimental results on procedural learning of visual sequences, of mirror drawing, of motor manual skills and of quick verification of central coherence, suggest that a basic neuropsychological dysfunction of procedural learning may be the central problem in DCD, with its frequent association to social communication disorder. This basic PL dysfunction seems to be intrinsic to DCD and independent of attention deficit: the experiments took account of attention deficit by considering a group of subjects with ADHD alone.

A limitation of the above experimental studies is that the tasks were highly specific. Similar studies with larger samples, with more diverse and ecological tasks, and with greater number of trials (to justify the assumption of long-term learning), are necessary.

Children with DCD can improve their motor and cognitive performance by repetition. Therefore, we suggest that this developmental condition does not imply an absolute inability, but a poor natural disposition, to learn motor and/or cognitive facilitating strategies. Assuming, as indicated by the research findings, that the core dysfunction lies in automation, an appropriate approach to help affected children would be to base intervention on repetition of the skills needed by each individual patient in his or her everyday ecological context and taking account of personal motivations and preserved abilities (for example, language for auto-instructions).

The persistent nature of DCD in around one-half of individuals first diagnosed in childhood (Cantell et al., 1994) emphasizes the importance of occupational therapy intervention in youth. The majority of approaches to intervention fit into two main categories. The “process or deficit approaches” aim to remedy some underlying process deficit with intervention targeted at a neural structure (Polatajko and Cantin, 2005). By contrast, the “functional skill approaches” work on teaching the activities of daily living that the child needs to be able to carry out. Recent meta-analyses demonstrate that the latter category of approaches produces the best therapeutic effect (Blank et al., 2012; Smits-Engelsman et al., 2013). Intervention designs should be addressed not only to the training of neurophysiological procedural circuitry but also to respond to motivations of each subject and to enhance generalization of newly acquired skills and good habits for managing significant cues of daily life, social relationships, and schooling (Polatajko and Cantin, 2005; Sugden and Dunford, 2007). The P4C model (Missiuna et al., 2011) emphasizes the partnership of the occupational therapist with educators and parents to change the life and daily environment of a child; the model focuses on capacity building through collaboration and coaching in context and includes whole class instruction, dynamic performance analysis, and monitoring response to intervention.

Neurobiological habits can be viewed as constrictions of dispositional resources of the agent. Such a perspective on operational habits is, perhaps, more appropriate for so-called “bad” or pathological habits, i.e., obsessions, tics, movement disorders etc. In this article, however, we have emphasized a positive, healthy view of habits because the functions of the human brain are precisely orchestrated on the basis of a huge number of beneficial automatisms that allow us to perform fluently the complex cognitive and motor activities of daily life. Psychoeducation can help young people suffering from DCD to become physically more adept and to liberate their potential for complex thinking, for planning of practical actions and for evaluating the social appropriateness of their behavior.

### Conflict of interest statement

The authors declare that the research was conducted in the absence of any commercial or financial relationships that could be construed as a potential conflict of interest.
